# Exploring Neuronal Vulnerability to Head Trauma Using a Whole Exome Approach

**DOI:** 10.1089/neu.2019.6962

**Published:** 2020-08-14

**Authors:** Omar Ibrahim, Heidi G. Sutherland, Neven Maksemous, Robert Smith, Larisa M. Haupt, Lyn R. Griffiths

**Affiliations:** Genomics Research Centre, Institute of Health and Biomedical Innovation, School of Biomedical Science, Queensland University of Technology (QUT), Kelvin Grove, Queensland, Australia.

**Keywords:** concussion, genetics, head trauma, ion channel, neurotransmitters

## Abstract

Brain injuries are associated with oxidative stress and a need to restore neuronal homeostasis. Mutations in ion channel genes, in particular *CACNA1A,* have been implicated in familial hemiplegic migraine (FHM) and in the development of concussion-related symptoms in response to trivial head trauma. The aim of this study was to explore the potential role of variants in other ion channel genes in the development of such responses. We conducted whole exome sequencing (WES) on16 individuals who developed a range of neurological and concussion-related symptoms following minor or trivial head injuries. All individuals were initially tested and shown to be negative for mutations in known FHM genes.

Variants identified from the WES results were filtered to identify rare variants (minor allele frequency [MAF] <0.01) in genes related to neural processes as well as genes highly expressed in the brain using a combination of *in silico* prediction tools (SIFT, PolyPhen, PredictSNP, Mutation Taster, and Mutation Assessor). Rare (MAF <0.001) or novel heterozygous variants in 7 ion channel genes were identified in 37.5% (6/16) of the cases (*CACNA1I, CACNA1C, ATP10A, ATP7B, KCNAB1, KCNJ10,* and *SLC26A4*), rare variants in neurotransmitter genes were found in 2 cases (*GABRG1* and *GRIK1*), and rare variants in 3 ubiquitin-related genes identified in 4 cases (*SQSTM1*, *TRIM2*, and *HECTD1*).

In this study, the largest proportion of potentially pathogenic variants in individuals with severe responses to minor head trauma were identified in genes previously implicated in migraine and seizure-related autosomal recessive neurological disorders. Together with results implicating variants in the hemiplegic migraine genes, *CACNA1A* and *ATP1A2*, in severe head trauma response, our results support a role for heterozygous deleterious mutations in genes implicated in neurological dysfunction and potentially increasing the risk of poor response to trivial head trauma.

## Introduction

Neuronal and synaptic ion channels, which include calcium, potassium, and sodium channels, ATPase transporters, and solute carriers, control the flow of ions and neurotransmitters through cellular membranes.^1^ In addition to their normal functions, they are crucial for restoring neuronal homeostasis in the aftermath of a head impact.^2^ Ion channelopathies, a wide range of dysfunctions related to ion channels and ion transporters, have long been documented as one of the main causes of several neurological disturbances. The calcium channel gene, *CACNA1A,* has been identified as a key gene in which mutations can cause familial hemiplegic migraine (FHM), along with several other FHM-related ion channel genes (*ATP1A2*, *SCN1A*). *CACNA1A*, which encodes the 1αpre-forming subunit of the Ca^2+^ voltage-gated Cav2.1 channel, has been shown to harbor mutations implicated in the development of an array of neurological disturbances and concussion-related symptoms (migraine, epilepsy, coma, and cerebral edema) following minor or trivial head trauma.^3–5^ Recently, mutations in the *ATP1A2* gene have also been found to be present in individuals of a large family with an over-representation of concussion incidents.^6^ Maksemous and colleagues^7^ additionally recently reported 8 *ATP1A2* variants in individuals who show severe responses to trivial head trauma. In addition, head trauma was found to start myriad neurological symptoms in individuals with variants in *ABCD1*, an X-linked adrenomyeloneuropathy-causing gene, which is implicated in cellular peroxisome functioning.^8^

These mutations can change the functionality and structure of ion channels, leading to abnormal responses to minor trauma or mild traumatic brain injury (mTBI).^9^ Additionally, ion channels are suggested to play a role in the development of acquired neurological dysfunction (e.g., epilepsy) following environmental disturbances (e.g., head injury or trauma).^10^ Further evidence suggests that genotype, particularly of neuronal genes including *KIAA0319*,^11^ *BDNF*,^12–14^ and *APOE*^15,16^ plays a role in response to head injury and recovery from mTBI.

Head injury or impact leads to cellular distortion and changes in membrane permeability, which contributes to an acute imbalance in extracellular ion gradients, in particular, K^+^, which is expelled into the extracellular space.^17,18^ Glutamate and other excitatory neurotransmitters are then released by neurons, likely due to the imbalance caused by K^+^ efflux, paired with Na^+^ and Ca^2+^ influx into the neurons, with the resulting ion imbalances contributing to local and downstream neuronal depolarization.^19^ Cellular-level homeostasis can be re-established through ion pump activation, requiring high adenosine triphosphate (ATP) expenditure and energy, a process that leads to higher neuronal oxidative stress.^18^ This energy depletion is characterized by hyperglycolysis (an above-average increase in glucose utilization^20^ and interference of Ca^2+^ ions with cellular and mitochondrial functioning. The high influx of Ca^2+^ following a TBI has been linked to long-term neuronal dysfunction,^21^ leading to an array of symptoms, with headache and migraine being the most common presentation.^22^

Although mutations in the calcium channel gene (*CACNA1A*) have been shown to affect neurological response to head trauma in carriers,^4,23–25^ several cases have occurred of patients presenting with similar reactions to head trauma with no mutations present in *CACNA1A*. In a cohort of patients referred for FHM genetic testing following severe reactions to trivial head trauma, the majority of individuals were shown to be negative for variants in FHM genes. Post-head injury symptoms include migraine, and cognitive or affective changes.^26^ However, the current study is focused on somatic rather than cognitive or behavioral symptoms. Based on the crucial role ion channels and neurotransmitters play in response to head trauma, we hypothesize that underlying mutations in ion channel and neurotransmitter genes might be implicated in the development of concussion symptoms following minor head trauma, similar to those observed in *CACNA1A* and *ATP1A2* cases. In this study we investigate the role of rare and functional variants in other ion homeostasis genes in relation to neurological symptoms following minor head trauma.

## Methods

### Cohort

Over 300 patients have been referred to the Genomics Research Centre (GRC) Diagnostic Clinic for HM diagnostic testing. Twenty-four patients reported episodes of confusional migraine, and other concussion-related symptoms after mild head trauma including loss of consciousness, seizure, amnesia and/or edema noted in their clinical descriptions. Although their presentation was heterogeneous, all individuals were identified to have head trauma or injury prior to the onset of their neurological symptoms. The 24 suspected case patients also had relevant symptoms as described in the International Headache Society (IHS) criteria for HM.

As reported in Maksemous and colleagues,^7^ 8 of the 24 case patients presenting with concussion-related symptoms had likely pathogenic variants in *ATP1A2*. The remaining 16 individuals had no deleterious or pathogenic mutations in FHM genes, prompting the whole exome sequencing (WES) approach used in this study. Ethical approval was granted for this study with samples obtained through the diagnostic facility of the GRC (Queensland University of Technology [QUT] Approval Number 1400000748). A summary of the clinical notes provided for the16 individuals examined in this study is provided in Table 1.

**Table 1.** Clinical Notes for 24 Cases Referred for Diagnostic Testing of Suspected Hemiplegic Migraine with Notable Varied Neurological Dysfunctions after Minor and/or Trivial Head Trauma

Case ID	Age	Sex	Clinical notes
R170	10–18	Male	Confusional migraine following minor head injury
R211	10–18	Male	Severe migraine and ataxia following head injury
R259	10–18	Female	Netball hit to right side of temple. Patient described surroundings going black and then quickly recovering. A soccer game made them more confused and presented to ER with hemiplegia. A background of headaches, unilateral throbbing, which increase upon sitting up, which typically occur in the morning and are a little bit unpredictable. Photophobia, noise sensitivity, and nausea reported. No other neurological phenomena, i.e., aura reported. Both maternal and paternal aunts have bad migraines; however, neither parents nor siblings, nor anyone else in the extended family suffer from migraines.
R117	0–10	Female	Recurrent episodes of pallor and vomiting following minor head injuries. Slurred speech with inappropriate words. Mild biparietal headache on most days since the injury; these headaches usually last for several minutes and resolve spontaneously. Consistent noise sensitivity and light sensitivitiy occurred for 3 weeks post-injury. Mother has a history of migraines but there is no other significant family history of neurological problems.
R118	0–10	Male	Catastrophic cerebral edema following trivial injury; mother has had hemiplegic migraines.
R197	10–18	Male	Migraine on minimal trauma, has two episodes of confusion and headache post rugby games.
R150	0–10	Female	Ischaemic stroke after mild head injury.
R171	10–18	Male	Acute confusional migraine after head injury.
R120	0–10	Male	Severe bleed following suspected minor fall. Only a relatively minor fall, but was followed by malignant cerebral edema and a relatively small subdural bleed with herniation within half an hour of the fall; there is no history or family history of migraine.
R240	10–18	Female	Family history of migraine. Netball hit head, lead to headache and confusion.
R110	10–18	Male	Repeated attacks of “concussion” after minor head trauma. After minor head trauma he develops a “migraine like” episode with slurred speech, diplopia, headache, and vomiting.
R111	30–50	Male	Similar to son, case patient R110.
R167	18–30	Male	Head injury induced migraine.
R206	18–30	Male	Left-sided numbness following a concussion.
R222	10–18	Male	Multiple episodes in a few months of visual disturbance, headache, and vomiting following trivial head trauma, most recently from hitting head against a player's chest at basketball.
R256	10–18	Female	An episode of stroke related to minor head trauma, seizure disorder, and episodic ataxia.

### DNA preparation and WES

DNA was extracted from blood samples using Qiagen DNeasy kits as per the manufacturer's instructions. Next-generation sequencing (NGS) libraries for WES were constructed using Ion AmpliSeq^TM^ Exome RDY Library Kits (Thermo Fisher Scientific) according to the manufacturer's protocol. The Ion Chef was used to load sample libraries (barcoded fragments of 200 base pairs [bp]) onto P1 chips using Ion P1^TM^ Hi-Q TM Chef Kit protocols. WES was performed on the Ion Proton (Thermo Fisher Scientific) instrument using default settings for Ion AmpliSeq Exome RDY - IC Kit 4x2.

### Statistical analysis

**FIG. 1.**
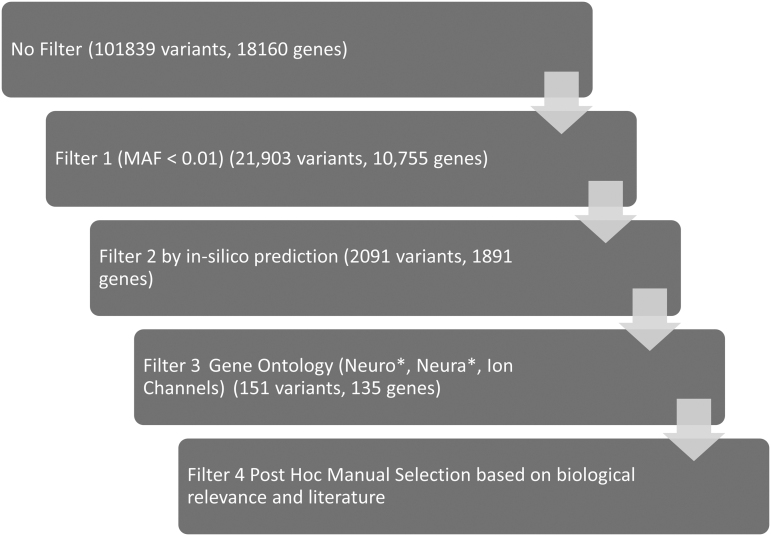
Variant filtering pipeline to explore relevant variants in patients with severe reaction in trivial head trauma (*n* = 16) using Ion Reporter software.

Following WES, the Ion Torrent Server was used to generate quality metrics, align reads to the Human Genome 19 (hg19), and the Ion Torrent Variant Caller (TVC) was used to call sequence variants. Whole exomes of the 16 patients negative for HM gene mutations were analyzed for genes implicated in known head-trauma-related conditions, followed by analysis, then all genes related to ion channels, or neuronal functions. Ion Reporter software (version 5.2) was used to explore and filter variants based on minor allele frequency (MAF), gene ontology, and functional scores. A summary of the filtering process and the corresponding genes and variant numbers is provided in Figure 1.

*In silico* prediction tools were then used to investigate the pathogenicity of the mutations. Two sets of tools were used, training-based algorithms (PolyPhen-2 and Mutation Taster) and non-training-based algorithms (SIFT and Mutation Assessor), to avoid results based solely on machine learning tools, which are prone to over-fitting.^27,28^

Mutation Assessor, an *in silico* prediction tool based on protein function, was used to explore the potential impact of amino acid (AA) changes on protein structure. Mutation Assessor produces functional impact scores (FI), wherein scores below 0.8 have a neutral impact, scores between 0.8 and 1.9 have a low impact, scores between 1.9 and 3.5 have a medium impact, and scores higher than 3.5 have a high impact.^29^ PredictSNP is a suitable tool for assessing the potential pathogenicity of variants, as it compiles the data of 8 of the top established prediction models and metrics (MAPP, nsSNPAnalyzer, PANTHER, PhD-SNP, PolyPhen-1, PolyPhen-2, SIFT, and SNAP^30^) to produce a percentage of predicted deleterious effect or non-pathogenicity as an average of all the tool prediction scores. Whereas PredictSNP incorporates PolyPhen as part of the prediction matrix, the PolyPhen total score is more relevant to this analysis. Consequently, using PolyPhen as a complementary score to SIFT in the first step through Ion Reporter is a stand-alone *a priori* filtering process that is not related to the overall score of PredictSNP. Mutation Taster, which predicts how potentially disease-causing a variant is,^31^ was used in conjunction with PredictSNP to establish a more detailed understanding of the effects of certain mutations identified as AA sequence changes, frame shifts, and splice sites.

All reported mutations were confirmed via Sanger Sequencing. Variant relevance was determined based on: 1) biological relevance, such as the expression of the gene in the brain, as determined by the National Center for Biotechnology Information [NCBI] gene database; 2) the limited number of individuals with the variant in other databases such as dbSNP and gnomAD; 3) existing evidence of the involvement of the variant or the gene in a relevant neurological disorder or pathology; and 4) evidence of involvement of the gene in a relevant biological process (i.e., response to concussion or neuroprotective factors).

To increase the robustness of the analysis, the 16 exomes were analyzed using the in-house variant browser pipeline, VCF-DART.^32^ The pipeline utilizes predetermined gene tiers (see Supplementary Appendix S1) as well as scores from various *in silico* prediction tools to filter variants of interest. The genes in those tiers were selected from established genetic diagnostic tests for several neurological disorders, with the last tier showing all variants that are not present in previous tiers, thus reducing the likelihood of missing any relevant variants in genes not selected in the tiers. Further, the analysis was conducted with other neurological exomes analyzed from epilepsy, CADASIL syndrome, and mTBI patients with no severe responses to trivial head trauma.

## Results

To identify variants in ion-channel and neuronal genes that might be involved in concussion response, WES was performed in 16 individuals who were referred for diagnostic testing after a severe response to a minor head trauma, but for whom no likely pathogenic variants were detected by full exonic sequencing of the known FHM genes. Quality metrics were above the accepted thresholds^33^ for all samples assayed, as detailed in Table 2. Samples had an average number of reads of 33x10^6^, with a minimum of 21x10^6^ reads. The average mean coverage depth was 99X across all samples, with a minimum coverage depth of 61X. Ion Torrent exome fragments are 200 bp long with an average read length in the samples 111 bp, with a range of 18 and standard deviation (SD) of 5.2, indicating an acceptable level of fragment integrity. Samples had an average of 37,550 called variants and a minimum of 35,600 variants. QC metrics per sample are reported in Table 2.

**Table 2.** Quality Metrics of WES by Ion Proton

Case ID	Mapped reads (millions)	Number of variants	Mean read length (bp)	Mean depth
R170	22.32	36432	177	61.7
R211	36.67	38092	192	112.9
R259	32.1	37658	190	96.71
R117	37.22	38028	190	111
R118	35.24	37166	188	100
R197	38.27	38119	R111	112.7
R150	21.7	38563	R111	65.6
R171	32.44	37356	R110	95.5
R120	47.17	37912	187	140
R240	40.3	37635	192	123
R110	26.6	37431	189	82.34
R111	30.8	37451	187	94.8
R167	29	37394	193	88.4
R206	27.8	37595	181	79.5
R222	41.1	38347	174	114.7
R256	39.3	35628	191	119.5
Mean	33.626875	37550.438	R111.75	99.896875
SD	6.8413958	699.85445	5.2141634	20.537952
Min.	21.7	35628	174	61.7

SD, standard deviation.

Ion Reporter has an ontology filter, wherein gene expression, function, and gene families can be used to filter genes. Following our hypothesis, which is based primarily on ion channel genes, we included the following gene ontologies in our analyses: neural, neurological, brain, ion channel, and neuro*. To identify rare and novel mutations, only variants with MAF below 0.001 were included. SIFT and PolyPhen-2, two complementary pathogenicity prediction tools, were used to filter variants in Ion Reporter to ensure that the mutations included were of a certain level of functional impact. It is worth mentioning that only one variant (*CACNA1C*p.Ile662Leu) was predicted to be benign by Polyphen, which was reflected by the lowest deleterious prediction score in Table 3a (82%). However, as all other *in silico* tools predicted this variant to be damaging, it was still included. Each case had an average of 25 filtered variants. The 16 filtered Variant Caller Files (VCFs) were screened for variants in genes in common. However, as none were identified, each case was assessed individually to identify gene variants most likely to contribute to poor response to head trauma as outlined below.

**Table 3A.** Predicted Deleteriousness by Several in Silico Tools for the Variants Found in Ion Channel Genes

Case ID	Gene	Trancript	Variant/AA change	SIFT	PolyPhen	PredictSNP2	Mutation Taster	Mutation Assessor (FI score)
R170	*ATP10A*	NM_024490.3	c.2642C>T/p.Ala881Val	D	Probably damaging	87% D	D/AA/Protein/SS	High (3.98)
R211	*ATP7B*	NM_000053.3	c.2383C>T/p.Leu795Phe	D	Probably damaging	82% D	D/AA/Protein/SS/Known potential disease mutation (HGMD CM970141)	Medium (3.35)
R211	*CACNA1I*	NM_021096.3	c.331C>G/p.Arg111Gly	D	Probably damaging	87% D	D/AA/Protein/SS	NA
R259	*CACNA1C*	NM_199460.2	c.1984A>C/p.Ile662Leu	D	B	82% D	D/AA/Protein/SS	NA
R117	*KCNJ10*	NM_002R120.4	c.52C>T/p.Arg18Trp	D	Possibly damaging	87% D	D/AA/Protein/SS	Medium (2.11)
R118	*KCNAB1*	NM_172160.2	c.749C>G/p.Ala250Gly	D	Probably damaging	87% D	D/AA/Protein/SS	Medium (2.915)
R197	*SLC26A4*	NM_000441.1	c.412G>A/p.Val138Ile	D	Probably damaging	87% D	D/AA/Protein/SS	Medium (3.215)

AA, amino acid; FI, functional impact.

**Table 3B.** Predicted Deleteriousness by Several in Silico Tools for the Variants Found in Neurotransmitter Genes

Case ID	Gene	Transcript	Variant/AA change	SIFT	PolyPhen	PredictSNP2	Mutation Taster	Mutation Assessor (FI score)
R150	*GABRG1*	NM_173536.3	c.137A>G/p.Asp46Gly	D	Probably damaging	87% D	D/AA changes/Protein Features/SS changes	Medium (2.11)
R171	*GRIK1*	NM_000830.4	c.1282A>T/p.Asn428Tyr	D	Probably damaging	87% D	D/AA/Protein/SS	Medium (3.43)

AA, amino acid; FI, functional impact.

**Table 3C.** Predicted Deleteriousness by Several in Silico Tools for the Variants Found in Ubiquitin Genes

Case ID	Gene	Trancript	Variant/AA change	SIFT	PolyPhen	PredictSNP2	Mutation Taster	Mutation Assessor (FI score)
R120	*TRIM2*	NM_015271.3	c.158G>C/p.Cys53Ser	D	Probably damaging	87% D	D/AA/Protein/SS	High (4.6)
R240	*HECTD1*	NM_015382.3	c.5000G>A/p.Arg1667His	D	Probably damaging	87% D	D/AA/Protein/SS	Low (1.83)
R110, R111	*SQSTM1*	NM_003900.4	c.1210A>G/p.Met404Val	D	Possibly damaging	87% D	D/AA/Protein/SS	Medium (2.98)

AA, amino acid; FI, functional impact.

**Table 3:** A–C. Key

SIFT	PolyPhen	PredictSNP2	Mutation Taster
T = Tolerated		D = Deleterious	D = Disease causing
D = Deleterious			AA changes = Amino acid changes
DL = Deleterious low confidence	B = Predicted benign		Protein = Protein feature might be affected
			SS = splice site changes

Rare and novel variants were found in 12 cases, including in genes encoding potassium channels *(KCNJ10* and *KCNAB1*), calcium channels (*CACNA1I* and *CACNA1C*), ATPases (*ATP10A* and *ATP7B*), Solute Carriers (*SLC26A4*), and neurotransmitter receptors (*GABRG1* and *GRIK1*). Further, in 4 of the cases, although no relevant variants in ion channel or neurotransmitter genes were found, variants in ubiquitin-related genes (*SQSTM1*, *HECTD1*, and *TRIM2*), which were included as part of the neuronal ontology filter, were detected and are of potential interest. MAFs of each of the identified variants from the databases are summarized in Table 4.

**Table 4.** Minor Allele Frequencies (MAFs) of Variants Deemed Relevant to Symptoms by WES Analysis

Case ID	Gene	Variant	gnomAD	TOP-MED count
R170	*ATP10A*	rs142704035	19 in 277134 (0.00006)	9
R211	*ATP7B*	rs751710854	7 in 246244 (0.00002)	
R211	*CACNA1I*	rs751729397	4 in R117482 (0.00001)	
R259	*CACNA1C*	chr12:2690844	_	
R117	*KCNJ10*	rs138457635	97 in 277144 = 0.0003500	61
R118	*KCNAB1*	No rs 3:156232893 C / G	1 in 239230 (0.000004)	
R197	*SLC26A4*	rs111033199	44 in 276848 (0.0001)	26
R150	*GABRG1*	rs759786658	4 in 274046 (0.00001)	2
R171	*GRIK1*	rs757997768	1 in 246130 (0.000004)	2
R120	*TRIM2*	No rs chr4:154191614G>C	_	
R240	*HECTD1*	rs371260055	13 in 276216 (0.00004)	
R110, R111	*SQSTM1*	rs771966860	4 in 246230 (0.00001)	2

WES, whole exome sequencing.

Scores from *in silico* prediction tools are reported in Table 3 for all the variants. All mutations identified via WES were predicted to be over 80% deleterious by predictSNP2 and predicted to be disease-causing (not a polymorphism) by Mutation Taster. Despite the variation in symptoms among the cases included in this study, the cohort can be categorized into four groups of main symptomatology: migraine, light and noise sensitivity, hemiparesis or hemiplegia, and cerebral edema or stroke. Using an alternative variant explorer (VCF-DART)^32^ produced results identical to those reached using Ion Reporter. This was assessed via exploring the variants with MAF less than 0.01 and predicted to be deleterious in the gene lists of Supplementary Appendix S1. In the 2 cases where the most relevant variant to the presentation was not in the gene lists, it was identified in the last tier where variants predicted to be highly deleterious in any additional genes are presented.

Of the 7 case patients who developed migraine following minor head injuries, 2 had ATPase-related variants. The variant in *ATP10A* has a high predicted functional impact and has an MAF of 0.00006, whereas the *ATP7B* variant has a medium predicted functional impact and has an even lower MAF of 0.00002. In the related (father and son) cases (R110 and R111), a Sequestome 1 (*SQSTM1*) mutation was identified (MAF = 0.00001). Another case patient, R197, was found to have an *SLC26A4* mutation, which is known as a pathogenic variant for Pendred syndrome, according to Human Gene Mutation Database (HGMD) and dbSNP (MAF = 0.0001). Finally, a glutamate receptor mutation (*GRIK1*), found in case patient R171, was found in one other individual only, in gnomAD (MAF = 0.000004).

There were 2 case patients who developed light and noise sensitivity following their minor head injuries. The first one (R259) had a *CACNA1C* mutation that is novel. The other case patient (R117) had a mutation in *KCNJ10*, which has been reported as a variant of uncertain significance for the autosomal recessive Seizures, Sensorineural deafness, and Ataxia, Mental retardation, and Electrolyte imbalance (SeSAME) syndrome and has a slightly higher MAF (0.0003) than the rest of the variants found in this cohort.

A third group presented with cerebral edema and ischemic stroke following head injuries. One case patient had a novel variant in *TRIM2*, one had a *GABRG1* variant with a MAF of 0.00001, and one had a *KCNAB1* variant that does not have an assigned reference SNP ID number on dbSNP.

## Discussion

In this study, we hypothesized that ion channel and neurotransmitter genes would harbor rare (MAF ≤0.001) deleterious mutations in individuals who developed concussion-related symptoms following mostly trivial head trauma. Among the 16 individuals screened by WES in this study, 12 case patients were identified to have variants predicted to be deleterious in other neuronal genes. Of the latter, deleterious variants either implicated in autosomal recessive neurological disorders or located in genes in which other mutations cause a neurological disorder were observed in 8 of 12 cases. Therefore, we propose that, similar to some *CACNA1A* and *ATP1A2* mutation carriers, individuals with deleterious variants in other neuronal genes, particularly those involved in ion homeostasis, may lead to an increased vulnerability to head trauma. This premise of genetic risk modulating the severity of acquired neurological dysfunction has already been demonstrated in animal models of epilepsy.^34^ As such, it follows that this hypothesis might also suit the case of neuronal vulnerability to head trauma.

### Ion homeostasis genes and pathways

Functional studies of FHM2 (*ATP1A2*) mutations in transgenic knock-in mice suggest that collectively, causal ion-channel mutations facilitate cortical spreading depression (with similar events implicated in concussion) as a result of defective removal of glutamate by glial cells, causing a similar downstream net effect as seen with FHM1 mutations.^35,36^ The established involvement of the Na^+^/K^+^-ATPase in the processes that follow head trauma as well as other neurological disorders supports our neuronal vulnerability hypothesis, and a potential role for ion channel genes in modulating concussion-related symptoms. In our cohort of individuals with severe response to minor head trauma, we identified variants predicted to be deleterious in genes related to ATPase, calcium and potassium channels, and other solute carriers.

### ATPase genes

Two ATPase-related genes*, ATP10A* and *ATP7B,* were identified as candidates in our cohort. *ATP10A* encodes a catalytic component of the P4-ATPase complex, which has a flippase activity influencing lipids in plasma membranes,^37^ whereas *ATP7B* encodes copper-binding domains and transmembrane copper transporters.^38^

Variants in *ATP10A,* another candidate gene in our cohort, have been found in individuals with autism (which share pathways with migraine,^39,40^ and Angelman syndrome.^41^ Russo and colleagues^40^ suggest that *ATP10A* imprinting is linked to migraine with aura (MA) through a locus in the 15q11-q13 region. In our study, case patient R170 with an *ATP10A* variant (p.Ala881Val) presented with a confusional migraine following minor head injury. Although this person might not routinely suffer from migraine, a stressor (head injury) may have triggered the *ATP10A* risk allele effect.

*ATP7B* encodes copper-binding domains and transmembrane copper transporters.^38^ The variant identified in this study in *ATP7B* (p.Leu795Phe) is located in transmembrane number 4 of the gene and is documented as a likely pathogenic allele for Wilson syndrome, an autosomal recessive copper metabolism disorder.^42^ Wilson syndrome is characterized by neurological dysfunction as the primary presentation in patients in their 20s and 30s.^38^ Although it is an autosomal recessive disorder, heterozygote mutations in *ATP7B* have also been found to affect related symptomatology in carriers.^43^ In addition, copper ion imbalance has also been linked to migraine and ataxia,^44,45^ with dysfunctional copper metabolism linked to axonal neuropathy.^46^ Case patient R211 in this study presented with a severe migraine and ataxia following a head injury, both of which are plausible phenotypes resulting from *ATP7B* mutations, potentially the result of an extreme stressor such as head trauma.

### Calcium channels

Voltage-gated calcium channels have been previously implicated in epilepsy, hemiplegic migraine, and schizophrenia^47,48^ with extracellular calcium levels influencing neurotransmitter secretion.^49^ *CACNA1A* remains the most investigated gene with regard to head trauma response. Mutations in *CACNA1A* demonstrate heterogenous neurological presentations, as exemplified by a family harboring the same mutation with varying symptomology including migraine, hemiplegia, coma, and progressive cerebellar ataxia.^50^ A novel *CANCA1C* (p.Ile662Leu) variant was detected in a case patient (R259) presenting with prolonged migraine attacks and photophobia following a minor hit on the head by a ball while playing a sport (netball). Interestingly, *CACNA1C* mechanisms have also been implicated in the survival of young neurons in the hippocampus and forebrains of animal models.^51^ Another case patient (R211) had a predicted deleterious rare variant (p.Arg111Gly) in the *CACNA1I* voltage-gated calcium channel gene and presented with a severe migraine and ataxia following head injury. *CACNA1I* has been implicated in neurological disorders as well as arthritis and schizophrenia.^52^ It should also be noted that this is the same case patient (R211) identified to have an *ATP7B* mutation, and these variants both may contribute to the response following trauma.

### Potassium channels

As discussed earlier, potassium channels play a crucial role in response to head trauma as well as several neurological disorders. We found a rare predicted-deleterious variant in *KCNJ10*, a gene previously implicated in symptom development and recovery following mTBI.^53^ *KCNJ10* encodes the potassium channel Kir4.1, an inwardly rectifying potassium channel, which restores negative resting potential mediating the clearing of extracellular glutamate.^54^ The case patient (R117) identified with a *KCNJ10* variant (p.Arg18Trp) presented with recurrent episodes of pallor and vomiting following minor head injuries. The patient identified sensitivity to loud noises and was sensitive to light for 3 weeks following the event. Additionally, the case patient's mother was noted to have a history of migraines, but there was no other significant family history of neurological problems. SeSAME-like syndrome has been reported in heterozygous carriers of other missense variants in *KCNJ10*^55^ and mutations in this gene have been found to cause epileptic encephalopathy.^56^

Another potassium channel gene identified with variants in our cohort was *KCNAB1,* which is strongly transcribed in the dorsolateral prefrontal cortex and encodes the potassium voltage channel Kv1.^57^ In this study, the case patient (R118) with a variant in *KCNAB1* (p.Ala250Gly) suffered catastrophic cerebral edema following a trivial injury. It is worth noting that *KCNAB1* is downregulated in peri-hematomal areas in the brain following hemorrhages, which usually leads to edema and secondary injuries.^58^ Hence, changes in potassium Kv1 voltage channel function due to an AA changing variant in *KCNAB1* may have contributed to the immediate neurological aftermath of the head injury.

### Neurotransmitters

Neurotransmission is essential to neuronal ion channel functioning, controlling excitability and inhibition based on the efflux and influx of neurotransmitters. Head injuries cause changes to neurotransmitter expression, which include but not limited to the gamma-aminobutyric acid (GABA)^59,60^ and glutamate^61^ receptors that perform a neuroprotective role in response to head injury.^62^

Neurotransmitters are disrupted following impact, during seizures and migraines and suppression of the GABA receptor subtype A is linked to post-stroke neuronal damage and ischemic death.^63^ Potentially pathogenic variants in genes involved in neurotransmitter pathways, *GABRG1* and *GRIK1*, were found in 2 cases in this study. GABA type-A receptor gamma1 subunit (*GABRG1*) encodes a protein that is part of the heteromeric pentameric ligand-gated ion channel family, highly expressed in the brain.^64^ Mutations in this gene have been implicated in epilepsy and seizure cases^65^ as well as in the aftermath of brain injuries, where significant changes in neurotransmitter GABA-related gene expression has been documented.^59^ In addition, GABA agonists have been found to protect against ischemia following injury.^63^ The case patient (R150) with a *GABRG1* variant (p.Asp46Gly) had developed an ischemic stroke following a mild head injury, which may indicate that any neuroprotective function conferred by *GABRG1* was impaired.

Another neurotransmitter, glutamate, whose levels rise dramatically following impact or stroke, is hypothesized to play a role in the aftermath of TBI. In particular, indiscriminate glutamate release leads to increased intracellular Ca^2+^ following concussion.^61^ Glutamate is also believed to contribute to secondary injuries through excitotoxicity.^66^ The *GRIK1* gene encodes an *N*-methyl-D-aspartate (NMDA) receptor and has been implicated in epilepsy-related neurological disorders^67^ as well as in migraine pathogenesis.^68^ The latter finding is compatible with the presentation of the case (R171) in which a *GRIK1* (p.Asn428Tyr) predicted deleterious variant was identified following the development of a confusional migraine after a minor head injury. With the principal of neuroprotection depending on a balance between excitatory (e.g., glutamate) and inhibitory (e.g., GABA) neurotransmitters,^69^ it follows that impairment of glutamate and GABA receptors may alter this balance and posit neurons at risk following stress (e.g., trauma).

The *SLC26A4* variant (p.Val138Ile) that we identified in case R197 is a variant reported to cause Pendred syndrome, an autosomal recessive disorder characterized by vestibular migraines as common comorbidity^70^ due to enlarged vestibular aqueducts and associated sensorineural dysfunctions.^71^ Interestingly, a case patient (R197) developed a migraine following minimal trauma and confusion and headache post rugby games, which may have provided a trigger if the patient was vulnerable from a deleterious allele.

### Ubiquitin genes

Sequestome 1 (*SQSTM1*) is the ubiquitin-binding protein p62, and mutations in this gene are implicated in frontotemporal dementia (FTD) and or amyotrophic lateral sclerosis (ALS).^72–75^ Several studies have identified that the mechanism with which p62 deficiency contributes to neuropathology is via impairment of complex 1 mitochondrial respiration.^76,77^ *SQSTM1* mutations have also been implicated in neurodegeneration with ataxia, dystonia, and gaze palsy, mostly linked to autophagy, and mitophagy in particular.^78,79^ The predicted pathogenic variant *SQSTM1* (p. Met404Val) was observed to be present in both a father (R111) and a son (R110) with a similar presentation, wherein after minor head trauma, both developed “migraine-like” episodes, with slurred speech, diplopia, headache, and vomiting. These neurological disturbances seem to overlap with those from the heterogeneous set of disorders implicating this gene. In particular, the proposed impairment of mitochondrial respiration could exacerbate neuronal oxidative stress following head trauma resulting in these episodes.

The *TRIM2* gene has a neuroprotective role and is an E3-ubiquitin ligase in proteasome-mediated degradation of target proteins. Loss of function mutations in this gene have been implicated in early-onset axonal neuropathy,^80^ and Charcot-Marie-Tooth autosomal recessive disorder.^81^ Thompson and colleagues^82^ demonstrated that *TRIM2* ubiquitination of a cell death mediator (Bim/Bcl-2) is one pathway for neuroprotection, and suppressing expression of *TRIM2* blocked this ubiquitination and any neuroprotective processes.

Further, Boone and colleagues^83^ found that *TRIM2* expression is suppressed in dying hippocampal cells following TBI. In this study, case patient R120 suffered severe bleeding following a minor fall, causing malignant edema and subdural bleeding. Impairment of the ubiquitin-proteasome system is implicated in neurodegeneration,^84^ with evidence from animal models of compromised *TRIM2* brain expression resulting in axonal swelling, accumulation of disorganized neurofilaments, and the formation of microtubules.^85^ Thus, we propose that a heterozygote mutation affecting the integrity of *TRIM2* ubiquitination properties might be exacerbated in the aftermath of an external trigger (fall). *HECTD1*, like *TRIM2*, is an E3-ubiquitin ligase, with mutations implicated in autism spectrum disorders and neurodegeneration. *HECTD1* plays a role in the development of the head mesenchyme and neural tube closure^86^ with mutations in this gene linked to neural tube defects.^87^ Case patient R240 with the identified *HECTD1* variant presented with confusion and headache following a minor head injury (netball).

It is not surprising that numerous genes in this study identified as candidates in response to head trauma are implicated in neurodegeneration.^88^ In a review by Sundman and colleagues,^89^ the occurrence of ALS in patients with mTBI was explored, finding higher odds ratios in cases of prior head trauma, suggesting a possible link. Here, we add to this hypothesis by proposing that neural vulnerability may involve a shared vulnerability to head trauma through neurodegeneration risk.

### Limitations

We acknowledge our lack of access to family members for this study, thus limiting our ability to establish these mutations as candidate causal variants. Further, the individuals examined in this study were referred specifically for suspected FHM symptoms. Hence, we acknowledge the heterogeneity of their presentations and we are unable to make any conclusions of susceptibility beyond our neuronal vulnerability hypothesis, which needs to be explored in larger cohorts of patients with mTBI and concussion. We also acknowledge the varied range of age in our population, and due to the sample size, it is difficult to determine if age at injury has any effect of variant pathogenicity. Our analysis targeted the inclusion of genes with neuronal-related ontology, and variants with a low MAF and high deleterious effect prediction scores from *in silico* prediction tools. Although this approach potentially leads to identifying variants with high penetrance, other more common, less deleterious variants potentially contribute to individual etiology.

## Conclusion

We have found rare and predicted-to-be-damaging variants in ion channel and neurotransmitter-related genes in addition to *CACNA1A* and *ATP1A2*, in individuals with severe responses to minor head trauma, implicating them in vulnerability to head trauma. These variants may cause functional changes to neurons, changing the influx of ions or efflux of neurotransmitters. Consequently, we hypothesize that although a heterozygote mutation does not lead to neurological symptoms under normal conditions, head trauma may act as a precipitant of disturbance. Therefore, concussion-related or migraine symptoms could develop in response to a disturbance precipitated by a head impact or trivial trauma in individuals who harbor heterozygous variants that cause autosomal recessive neurological disorders. As the evidence for ion channel involvement in head trauma increases, larger affected cohorts are required to be recruited and examined to confirm the relevance of each gene to specific symptoms subsets.

## Supplementary Material

Supplemental data

## Data accessibility

Sequencing data are available upon request, subject to the data sharing and transfer agreements of respective institutions.
