# Supplementary material for: Exploring Neuronal Vulnerability to Head Trauma Using a Whole Exome Approach
Source: J Neurotrauma. 2020 Aug 14;37(17):1870–9. doi: 10.1089/neu.2019.6962 (PMC7462038; doi:10.1089/neu.2019.6962)
Supplement: Supplemental data [file Supp_Appendix1.pdf]

Supplementary Data

Supplementary Appendix S1

Tier 1	Tier 2	Tier 3
ABCD1	*ABHD14B*	*ACAN*
ABCD1P2	*ABHD17AP2*	*ADAMTS2*
ABCD1P3	*APTR*	*ADAMTS9*
ABCD1P4	*ATP2B1*	*ANK3*
ABCD1P5	*KATNBL1P2*	*ANXA1*
ATP1A1	*LAPTM4B*	*Anxa2*
ATP1A1OS	*MAPT*	*APOE*
ATP1A2	*NACAD*	*ARHGAP20*
ATP1A3	*ABCA8*	*B3GNT7*
ATP1A4	*ABCB1*	*B4GALT2*
CACNA1A	*ABCB10*	*BCL11A*
CACNA1A	*ABCB10P1*	*CAMK4*
CACNA1B	*ABCB10P3*	*CDH13*
CACNA1C	*ABCB10P4*	*CHST5*
CACNA1C	*ABCB11*	*CHST6*
CACNA1D	*ABCB4*	*COLEC12*
CACNA1D	*ABCB5*	*DGKH*
CACNA1E	*ABCB6*	*DPP10*
CACNA1F	*ABCB7*	*DYSF*
CACNA1F	*ABCB8*	*ELMO1*
CACNA1G	*ABCB9*	*ESR1*
CACNA1H	*ABHD1*	*ESRRB*
CACNA1H	*ABHD10*	*FMOD*
CACNA1I	*ABHD11*	*GRIN2A*
CACNA1S	*ABHD12*	*KERA*
CACNA2D1	*ABHD12B*	*LIPC*
CACNA2D2	*ABHD13*	*LRRC7*
CACNA2D3	*ABHD14A*	*LRRN3*
CACNA2D4	*ABHD15*	*LUM*
FSCN1	*ABHD16A*	*LYPLAL1*
FSCN2	*ABHD16B*	*MAPK1*
FSCN3	*ABHD17A*	*NDN*
OBSCN	*ABHD17AP1*	*NMU*
PRRT1	*ABHD17AP3*	*NR4A2*
PRRT2	*ABHD17AP4*	*OGN*
PRRT3	*ABHD17AP5*	*OMD*
PRRT4	*ABHD17AP6*	*PAWR*
SCN10A	*ABHD17B*	*PCDH11X*
SCN10A	*ABHD17C*	*PDE7B*
SCN11A	*ABHD18*	*PLAT*
SCN11A	*ABHD2*	*PLEKHG1*
SCN1A	*ABHD3*	*PLG*
SCN1B	*ABHD4*	*PPP1R3B*
SCN2A	*ABHD5*	*PRELP*
SCN2B	*ABHD6*	*PROX1*
SCN3A	*ABHD8*	*RPS6KA1*
SCN3B	*ACAD10*	*S100A10*
SCN4A	*ACAD11*	*S100A4*
SCN4A	*ACAD8*	*SCARA3*
SCN4B	*ACAD9*	*SDCBP*
SCN5A	*ACADL*	*SEMA3A*
SCN5A	*ACADM*	*SERINC2*
SCN7A	*ACADS*	*SLC17A7*
SCN8A	*ACADSB*	*SLC26A4*

(*continued*)

Supplementary Appendix S1. (Continued)

Tier 1	Tier 2	Tier 3
SCN9A	*ACADVL*	*SMC2*
SCN9A	*AFG3L1P*	*SP6*
SCNM1	*AFG3L2*	*TCEB1*
SCNN1A	*AFG3L2P1*	*TGFB2*
SCNN1B	*ANO1*	*TRIM72*
SCNN1D	*ANO10*	*TSHZ2*
SCNN1G	*ANO2*	*TTLL7*
	ANO3	*TYRP1*
	ANO4	*UX2*
	ANO5	*ZDHHC21*
	ANO6	*ZIC5*
	ANO7	*CAPN1*
	ANO7P1	*CAPN2*
	ANO8	*CAPN3*
	ANO9	*CAPN5*
	ANOS1	*CAPN6*
	APTX	*CAPN7*
	ATCAY	*CAPN8*
	ATM	*CAPN9*
	ATMIN	*CAPN10*
	ATN1	*CAPN11*
	ATP2B2	*CAPN12*
	ATP2B3	*CAPN13*
	ATP2B4	*CAPN14*
	ATXN1	*CAPN15*
	ATXN10	*ADGB*
	ATXN1L	*CASP1*
	ATXN2	*CASP2*
	ATXN2L	*CASP3*
	ATXN3	*CASP4*
	ATXN3L	*CASP5*
	ATXN7	*CASP6*
	ATXN7L1	*CASP7*
	ATXN7L2	*CASP8*
	ATXN7L3	*CASP9*
	ATXN7L3B	*CASP10*
	ATXN8OS	*CASP12*
	BEAN1	*CASP14*
	C10orf2	*CASP16P*
	C10orf25	*IL1B*
	CA8	*IL1-RN*
	CCDC88C	*IL6*
	CDCA8	*IL8*
	COQ10A	*IL10*
	COQ10B	*PTGS2*
	COQ10BP2	*TNFA*
	COQ2	*CCBP2*
	COQ3	*CCL1*
	COQ4	*CCL2*
	COQ5	*CCL3*
	COQ6	*CCL4*
	COQ7	*CCL5*
	COQ8B	*CCL6*
	COQ9	*CCL7*
	CTDP1	*CCL8*

(*continued*)

Supplementary Appendix S1. (Continued)

Tier 1	Tier 2	Tier 3
	CWF19L1	*CCL9*
	DCTPP1	*CCL11*
	DNMT1	*CCL12*
	DNMT3A	*CCL13*
	DNMT3B	*CCL14*
	DNMT3L	*CCL15*
	EEF2	*CCL16*
	EEF2K	*CCL17*
	EEF2KMT	*CCL18*
	ELOVL4	*CCL19*
	ELOVL5	*CCL20*
	FGF14	*CCL21*
	FXN	*CCL22*
	GAPT	*CCL23*
	GATM	*CCL24*
	GLTPP1	*CCL25*
	GRID2	*CCL26*
	GRID2IP	*CCL27*
	GRM1	*CCL28*
	ITPR1	*CCR1*
	KATNA1	*CCR2*
	KATNAL1	*CCR3*
	KATNAL2	*CCR4*
	KATNB1	*CCR5*
	KATNBL1	*CCR6*
	KATNBL1P1	*CCR7*
	KATNBL1P3	*CCR8*
	KATNBL1P4	*CCR9*
	KATNBL1P5	*CCR10*
	KATNBL1P6	*CCRL2*
	KCNC1	*CX3CL1*
	KCNC2	*CX3CR1*
	KCNC3	*CXCL1*
	KCNC4	*CXCL2*
	KCND1	*CXCL3*
	KCND2	*CXCL4*
	KCND3	*CXCL5*
	LAPTM4A	*CXCL6*
	LAPTM5	*CXCL7*
	MAFG	*CXCL9*
	MATN1	*CXCL9*
	MATN2	*CXCL10*
	MATN3	*CXCL11*
	MATN4	*CXCL12*
	MIATNB	*CXCL13*
	NANOG	*CXCL14*
	NANOGNB	*CXCL15*
	NANOGNBP1	*CXCL16*
	NANOGNBP2	*CXCL17*
	NANOGNBP3	*CXCR3*
	NANOGP1	*CXCR4*
	NANOGP10	*CXCR5*
	NANOGP2	*CXCR6*
	NANOGP3	*CXCR7*
	NANOGP4	*DUFFY*
	NANOGP5	*IL8*
	NANOGP6	*IL8RA*
	NANOGP7	*IL8RB*
	NANOGP8	*ITGA4*
	NANOGP9	*ITGB1*
	NANOS1	*TREM1*
	NANOS2	*TREM2*

(*continued*)

Supplementary Appendix S1. (Continued)

Tier 1	Tier 2	Tier 3
	NANOS3	*TREM3*
	NOP56	*XCL1*
	PANO1	*XCR1*
	PAPOLG	*GRIN1*
	PDSS1	*GRIN2B*
	PDSS2	*GRIN2C*
	PDYN	*GRIN2D*
	POLG	*GRIN3A*
	POLG2	*GRIN3B*
	PPP2R2A	*GRIA1*
	PPP2R2B	*GRIA2*
	PPP2R2C	*GRIA3*
	PPP2R2D	*GRIA4*
	PPP2R2DP1	*TARDBP*
	PRKCG	*APP*
	RUBCN	*SNCA*
	RUBCNL	*SOD1*
	SACS	*FUS*
	SETX	*ACE*
	SFXN1	*ENPEP*
	SFXN2	*NOS1*
	SFXN3	*NOS2*
	SFXN4	*NOS3*
	SFXN5	*PHACTR1*
	SIL1	*COMT*
	SLC1A3	*MAOA*
	SPTBN2	*SLC1A2*
	STUB1	*SLC17A7*
	SYNE1	*SLC6A4*
	SYT14	*MTDH*
	SYT14P1	*AGT*
	TAPT1	*EDN1*
	TBP	*EDN3*
	TDP1	*BDNF*
	TGM6	*CALCA*
	TPP1	*AGTR2*
	TTBK2	*ADRA2A*
	TTPA	*EDNRB*
	TTPAL	*HTR1A*
	VAMP1	*HTR2A*
	VLDLR	*ADORA1*
	WWOX	*EDNRA*
	ZNF592	*PTGER2*
	OQ8A	*HTR1B*
		ABCC1
		ABCC8
		DUSPS
		TRPM2
		TRPM8
		CACN2B4
		TRPM4
		AQP4
		MMP2
		MMP3
		MMP9
		TSPO
